# *tert*-Butyl 2-de­oxy-4,5-*O*-isopropyl­idene-d-gluconate

**DOI:** 10.1107/S160053680803064X

**Published:** 2008-09-27

**Authors:** Sarah F. Jenkinson, K. Victoria Booth, Daniel Best, George W. J. Fleet, David J. Watkin

**Affiliations:** a Department of Organic Chemistry, Chemistry Research Laboratory, Department of Chemistry, University of Oxford, Oxford OX1 3TA, England; b Department of Chemical Crystallography, Chemistry Research Laboratory, Department of Chemistry, University of Oxford, Oxford OX1 3TA, England

## Abstract

The relative configuration of *tert*-butyl 2-de­oxy-4,5-*O*-iso­propyl­idene-d-gluconate, C_13_H_24_O_6_, an inter­mediate in the synthesis of 2-de­oxy sugars, was determined by X-ray crystallography, and the crystal structure consists of chains of O—H⋯O hydrogen-bonded mol­ecules running parallel to the *a* axis. There are two mol­ecules in the asymmetric unit. The absolute configuration was inferred from the use of d-erythrono­lactone as the starting material.

## Related literature

For background information, see: Granstrom *et al.* (2004 ▶); Izumori (2002 ▶, 2006 ▶); Rao *et al.* (2008 ▶); Yoshihara *et al.* (2008 ▶); Gullapalli *et al.* (2007 ▶); Jones *et al.* (2008 ▶). For related structures, see: Booth *et al.* (2008 ▶); Jenkinson, Booth, Gullapalli *et al.* (2008 ▶); Jenkinson, Booth, Yoshihara *et al.* (2008 ▶). For related literature, see: Görbitz (1999 ▶).
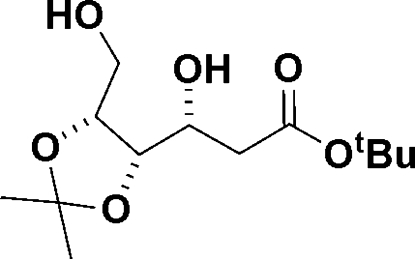


## Experimental

### 

#### Crystal data

C_13_H_24_O_6_
*M* *_r_* = 276.33
Triclinic, 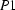

*a* = 5.9366 (2) Å
*b* = 11.1694 (5) Å
*c* = 12.7530 (6) Å
α = 113.413 (2)°
β = 100.3696 (19)°
γ = 93.783 (2)°
*V* = 754.42 (6) Å^3^
*Z* = 2
Mo *K*α radiation
μ = 0.10 mm^−1^
*T* = 150 K
0.50 × 0.05 × 0.05 mm

#### Data collection

Nonius KappaCCD diffractometer
Absorption correction: multi-scan (*DENZO*/*SCALEPACK*; Otwinowski & Minor, 1997 ▶) *T*_min_ = 0.73, *T*_max_ = 1.00 (expected range = 0.727–0.995)
11759 measured reflections
3441 independent reflections
2788 reflections with *I* > 2σ(*I*)
*R*_int_ = 0.045

#### Refinement

*R*[*F* ^2^ > 2σ(*F* ^2^)] = 0.040
*wR*(*F* ^2^) = 0.104
*S* = 0.97
3441 reflections
344 parameters
3 restraints
H-atom parameters constrained
Δρ_max_ = 0.29 e Å^−3^
Δρ_min_ = −0.37 e Å^−3^

### 

Data collection: *COLLECT* (Nonius, 2001 ▶).; cell refinement: *DENZO*/*SCALEPACK* (Otwinowski & Minor, 1997 ▶); data reduction: *DENZO*/*SCALEPACK*; program(s) used to solve structure: *SIR92* (Altomare *et al.*, 1994 ▶); program(s) used to refine structure: *CRYSTALS* (Betteridge *et al.*, 2003 ▶); molecular graphics: *CAMERON* (Watkin *et al.*, 1996 ▶); software used to prepare material for publication: *CRYSTALS*.

## Supplementary Material

Crystal structure: contains datablocks global, I. DOI: 10.1107/S160053680803064X/lh2696sup1.cif

Structure factors: contains datablocks I. DOI: 10.1107/S160053680803064X/lh2696Isup2.hkl

Additional supplementary materials:  crystallographic information; 3D view; checkCIF report

## Figures and Tables

**Table 1:** Hydrogen-bond geometry (Å, °)

*D*—H⋯*A*	*D*—H	H⋯*A*	*D*⋯*A*	*D*—H⋯*A*
O19—H22⋯O38^i^	0.85	2.03	2.861 (4)	165
O13—H49⋯O19^ii^	0.81	2.06	2.842 (4)	162
O39—H5⋯O18^iii^	0.83	1.98	2.771 (4)	157

Symmetry codes: (i) 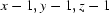; (ii) 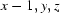; (iii) 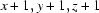.

**Fig. 1.**
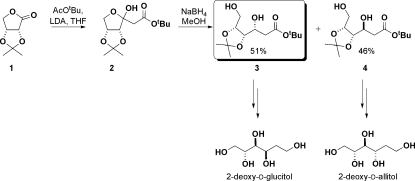
Synthetic scheme for the synthesis of the title compound.

**Fig. 2.**
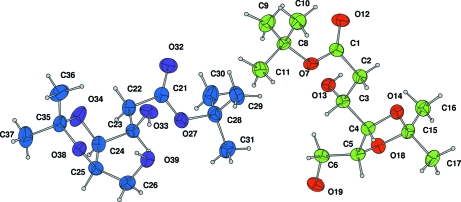
The title compound with displacement ellipsoids drawn at the 50% probability level. H atoms are shown as spheres of arbitary radius. There are two molecules in the asymmetric unit. Carbon atoms are shown in green (molecule 1) or blue (molecule 2) and oxygen atoms in red (molecule 1) or purple (molecule 2).

**Fig. 3.**
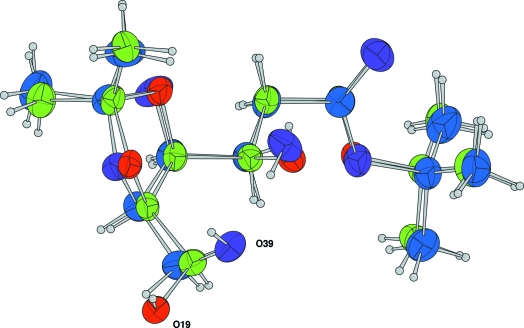
Overlay of the two molecules in the asymmetric unit.

**Fig. 4.**
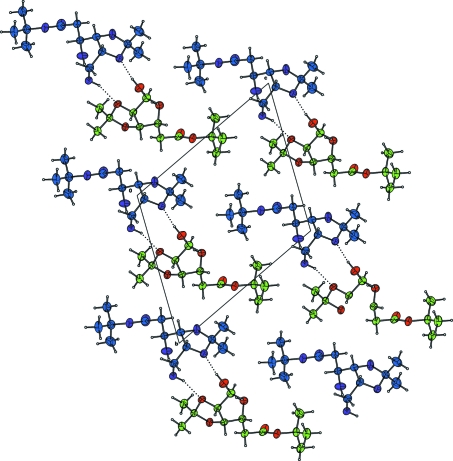
Packing diagram projected along the a-axis. Hydrogen bonds are shown by dotted lines.

**Fig. 5.**
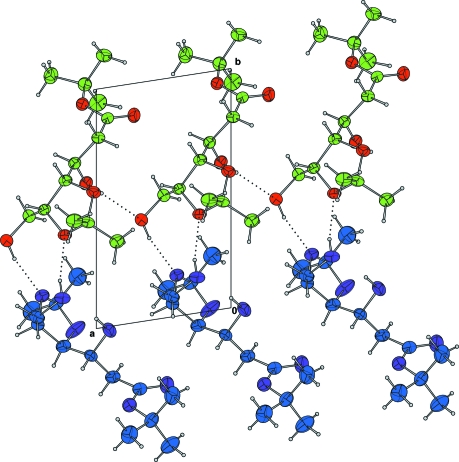
Packing diagram projected along the c-axis. The crystal structure consists of layers of hydrogen bonded molecules lying perpendicular to the c-axis.

## supplementary crystallographic information

### Comment

The technique of Izumoring (Izumori, 2002; Izumori, 2006; Granstrom *et al.*, 2004), the biotechnological interconversion of monosaccharides, has been seen to be generally applied to 1-deoxy-(Yoshihara *et al.*, 2008; Booth *et al.*, 2008; Jenkinson, Booth, Gullapalli *et al.*, 2008; Jenkinson, Booth, Yoshihara *et al.*, 2008; Gullapalli *et al.*, 2007) and methyl-branched sugars (Rao *et al.*, 2008; Jones *et al.*, 2008). In order to extend this methodology further, a series of 2-deoxy sugars were synthesized *via* the addition of lithium *tert*-butyl acetate to sugar lactones. Thus, lithium *tert*-butyl acetate was added to D-erythronolactone 1 and on reduction two compounds were obtained (Fig. 1). One product was crystalline and was unequivocally identified by X-ray crystallography to be the *tert*-butyl 2-deoxy-4,5-*O*-isopropylidene-D-gluconate (Fig. 2), with the absolute configuration being determined by the use of D-erythronolactone as the starting material.

The X-ray structure shows that there are two molecules in the asymmetric unit, these only differ in the orientation of the terminal hydroxyl groups O19 and O39 (Fig. 3) (torsion angles C4-C5-C6-O19 = 69.2° and C24-C25-C26-O39 = -170.4°). The remainder of the residues are very similar (Fig.3). After least squares fitting of the residues (excluding O19 and O39) against each other, the r.m.s. positional discrepancy is 0.1270 Å, the r.m.s. bond length discrepancy is 0.0082 degrees, and the r.m.s. torsion angle deviation is 5.5037 degrees. The molecules form hydrogen-bonded chains running parallel to the *a*-axis (Fig. 4, Fig.5). Only classic intermolecular hydrogen bonding has been considered.

### Experimental

The title compound was recrystallized from toluene: m.p. 345–347 K; [α]_D_^22^ +10.7 (*c*, 0.99 in CHCl_3_).

### Refinement

In the absence of significant anomalous scattering, Friedel pairs were merged and the absolute configuration was assigned from the starting material.

The relatively large ratio of minimum to maximum corrections applied in the multiscan process (1:1.37) reflect changes in the illuminated volume of the crystal. Changes in illuminated volume were kept to a minimum, and were taken into account (Görbitz, 1999) by the multi-scan inter-frame scaling (*DENZO*/*SCALEPACK*, Otwinowski & Minor, 1997).

The H atoms were all located in a difference map, but those attached to carbon atoms were repositioned geometrically. The H atoms were initially refined with soft restraints on the bond lengths and angles to regularize their geometry (C—H in the range 0.93–0.98, O—H = 0.82 Å) and *U*_iso_(H) (in the range 1.2–1.5 times *U*_eq_ of the parent atom), after which the positions were refined with riding constraints.

### Crystal data

**Table:**

C_13_H_24_O_6_	*Z* = 2
*M**_r_* = 276.33	*F*(000) = 300
Triclinic, *P*1	*D*_x_ = 1.216 Mg m^−^^3^
Hall symbol: P 1	Mo *K*α radiation, λ = 0.71073 Å
*a* = 5.9366 (2) Å	Cell parameters from 3172 reflections
*b* = 11.1694 (5) Å	θ = 5–27°
*c* = 12.7530 (6) Å	µ = 0.10 mm^−^^1^
α = 113.413 (2)°	*T* = 150 K
β = 100.3696 (19)°	Plate, colourless
γ = 93.783 (2)°	0.50 × 0.05 × 0.05 mm
*V* = 754.42 (6) Å^3^	

### Data collection

**Table:**

Nonius KappaCCD diffractometer	2788 reflections with *I* > 2σ(*I*)
graphite	*R*_int_ = 0.045
ω scans	θ_max_ = 27.6°, θ_min_ = 5.2°
Absorption correction: multi-scan (DENZO/SCALEPACK; Otwinowski & Minor, 1997)	*h* = −7→7
*T*_min_ = 0.73, *T*_max_ = 1.00	*k* = −14→14
11759 measured reflections	*l* = −16→15
3441 independent reflections	

### Refinement

**Table:**

Refinement on *F*^2^	Hydrogen site location: inferred from neighbouring sites
Least-squares matrix: full	H-atom parameters constrained
*R*[*F*^2^ > 2σ(*F*^2^)] = 0.040	*w* = 1/[σ^2^(*F*^2^) + (0.05*P*)^2^ + 0.04*P*], where *P* = [max(*F*_o_^2^,0) + 2*F*_c_^2^]/3
*wR*(*F*^2^) = 0.104	(Δ/σ)_max_ = 0.009
*S* = 0.97	Δρ_max_ = 0.29 e Å^−^^3^
3441 reflections	Δρ_min_ = −0.37 e Å^−^^3^
344 parameters	Extinction correction: Larson (1970), Equation 22
3 restraints	Extinction coefficient: 300 (50)
Primary atom site location: structure-invariant direct methods	

### Fractional atomic coordinates and isotropic or equivalent isotropic displacement parameters (Å^2^)

**Table:**

	*x*	*y*	*z*	*U*_iso_*/*U*_eq_	
C1	−0.0837 (4)	0.8713 (3)	0.3566 (2)	0.0306	
C2	−0.0148 (4)	0.7957 (3)	0.2427 (2)	0.0322	
C3	0.1598 (5)	0.7048 (3)	0.2533 (2)	0.0300	
C4	0.2288 (4)	0.6384 (3)	0.1362 (2)	0.0296	
C5	0.3854 (4)	0.5302 (2)	0.1211 (2)	0.0297	
C6	0.4988 (4)	0.5130 (3)	0.2286 (2)	0.0338	
O7	0.1033 (3)	0.94685 (18)	0.43945 (15)	0.0333	
C8	0.0854 (5)	1.0276 (3)	0.5600 (2)	0.0342	
C9	−0.0134 (6)	0.9420 (4)	0.6127 (3)	0.0497	
C10	−0.0575 (5)	1.1357 (3)	0.5601 (3)	0.0448	
C11	0.3368 (5)	1.0859 (3)	0.6218 (3)	0.0469	
O12	−0.2796 (3)	0.8666 (2)	0.37003 (19)	0.0434	
O13	0.0757 (3)	0.61392 (18)	0.29471 (16)	0.0330	
O14	0.0195 (3)	0.57145 (19)	0.04704 (15)	0.0335	
C15	0.0657 (5)	0.4490 (3)	−0.0322 (2)	0.0315	
C16	−0.1538 (5)	0.3507 (3)	−0.0776 (2)	0.0403	
C17	0.1621 (5)	0.4644 (3)	−0.1296 (2)	0.0408	
O18	0.2336 (3)	0.41137 (18)	0.04004 (16)	0.0325	
O19	0.6656 (3)	0.4251 (2)	0.20259 (18)	0.0391	
C21	0.6774 (5)	0.7195 (3)	0.8287 (2)	0.0356	
C22	0.8773 (5)	0.7868 (3)	0.9330 (2)	0.0357	
C23	1.0405 (4)	0.8902 (3)	0.9222 (2)	0.0306	
C24	1.2437 (5)	0.9472 (3)	1.0283 (2)	0.0350	
C25	1.4344 (5)	1.0537 (3)	1.0373 (2)	0.0327	
C26	1.4393 (5)	1.0846 (3)	0.9321 (2)	0.0373	
O27	0.7534 (3)	0.6588 (2)	0.73097 (16)	0.0386	
C28	0.5895 (5)	0.5818 (3)	0.6156 (2)	0.0400	
C31	0.7540 (6)	0.5357 (4)	0.5339 (3)	0.0531	
C30	0.4520 (6)	0.4661 (4)	0.6211 (3)	0.0553	
C29	0.4360 (6)	0.6706 (4)	0.5831 (3)	0.0533	
O32	0.4758 (4)	0.7182 (2)	0.8340 (2)	0.0513	
O33	0.9021 (3)	0.9849 (2)	0.90964 (19)	0.0433	
O34	1.1547 (4)	1.0087 (2)	1.13082 (17)	0.0549	
C35	1.2946 (5)	1.1313 (3)	1.2071 (2)	0.0377	
C36	1.1384 (6)	1.2284 (4)	1.2579 (3)	0.0572	
C37	1.4752 (6)	1.1154 (4)	1.2987 (3)	0.0593	
O38	1.4021 (3)	1.17115 (18)	1.13186 (15)	0.0334	
O39	1.2520 (3)	1.14885 (18)	0.90474 (16)	0.0354	
H21	0.0552	0.8664	0.2265	0.0350*	
H374	−0.1535	0.7491	0.1885	0.0343*	
H31	0.3032	0.7527	0.3023	0.0358*	
H41	0.3072	0.7113	0.1241	0.0318*	
H51	0.5123	0.5565	0.0937	0.0299*	
H61	0.5785	0.5972	0.2854	0.0415*	
H62	0.3842	0.4867	0.2676	0.0379*	
H91	0.0091	0.9931	0.6969	0.0748*	
H92	−0.1781	0.9090	0.5741	0.0753*	
H93	0.0708	0.8666	0.5969	0.0762*	
H101	−0.0695	1.1892	0.6383	0.0603*	
H102	0.0184	1.1924	0.5310	0.0619*	
H103	−0.2112	1.0937	0.5094	0.0604*	
H111	0.3490	1.1432	0.7056	0.0638*	
H112	0.3911	1.1360	0.5817	0.0621*	
H113	0.4303	1.0139	0.6121	0.0605*	
H161	−0.1335	0.2644	−0.1356	0.0533*	
H162	−0.2710	0.3893	−0.1118	0.0541*	
H163	−0.2018	0.3427	−0.0102	0.0533*	
H171	0.2104	0.3825	−0.1764	0.0583*	
H172	0.0439	0.4833	−0.1809	0.0615*	
H173	0.2932	0.5356	−0.0931	0.0603*	
H221	0.8135	0.8309	1.0028	0.0370*	
H222	0.9674	0.7230	0.9470	0.0375*	
H231	1.1005	0.8416	0.8441	0.0329*	
H241	1.3149	0.8725	1.0324	0.0438*	
H251	1.5871	1.0245	1.0503	0.0384*	
H261	1.5857	1.1453	0.9544	0.0459*	
H262	1.4308	1.0016	0.8639	0.0418*	
H311	0.6643	0.4781	0.4559	0.0697*	
H312	0.8410	0.6073	0.5269	0.0725*	
H313	0.8602	0.4848	0.5575	0.0711*	
H301	0.3512	0.4153	0.5456	0.0753*	
H302	0.3615	0.5007	0.6793	0.0752*	
H303	0.5565	0.4129	0.6424	0.0755*	
H291	0.3550	0.6232	0.5002	0.0783*	
H292	0.3233	0.6922	0.6327	0.0779*	
H293	0.5342	0.7508	0.5961	0.0786*	
H361	1.2311	1.3140	1.3097	0.0856*	
H362	1.0554	1.1946	1.3015	0.0842*	
H363	1.0313	1.2354	1.1954	0.0846*	
H371	1.5676	1.2009	1.3512	0.0897*	
H372	1.3954	1.0796	1.3409	0.0880*	
H373	1.5744	1.0553	1.2600	0.0883*	
H22	0.6069	0.3443	0.1745	0.0550*	
H37	0.9988	1.0407	0.9081	0.0730*	
H49	−0.0394	0.5617	0.2550	0.0476*	
H5	1.2694	1.2233	0.9597	0.0500*	

### Atomic displacement parameters (Å^2^)

**Table:**

	*U*^11^	*U*^22^	*U*^33^	*U*^12^	*U*^13^	*U*^23^
C1	0.0284 (13)	0.0241 (13)	0.0345 (13)	0.0024 (10)	0.0056 (10)	0.0082 (11)
C2	0.0343 (13)	0.0255 (13)	0.0318 (12)	0.0046 (10)	0.0077 (10)	0.0069 (11)
C3	0.0297 (12)	0.0274 (13)	0.0316 (12)	0.0024 (10)	0.0081 (9)	0.0109 (11)
C4	0.0343 (13)	0.0236 (13)	0.0312 (12)	0.0027 (10)	0.0103 (10)	0.0110 (11)
C5	0.0298 (12)	0.0228 (13)	0.0335 (12)	0.0009 (10)	0.0102 (10)	0.0079 (10)
C6	0.0298 (12)	0.0320 (14)	0.0391 (14)	0.0067 (11)	0.0087 (10)	0.0136 (11)
O7	0.0300 (9)	0.0307 (10)	0.0312 (9)	0.0033 (7)	0.0109 (7)	0.0030 (8)
C8	0.0352 (13)	0.0356 (15)	0.0266 (12)	0.0057 (11)	0.0112 (10)	0.0058 (11)
C9	0.0574 (19)	0.0531 (19)	0.0414 (15)	0.0020 (15)	0.0160 (14)	0.0213 (15)
C10	0.0477 (16)	0.0340 (16)	0.0424 (16)	0.0098 (13)	0.0092 (13)	0.0053 (13)
C11	0.0385 (16)	0.0500 (19)	0.0384 (14)	0.0013 (13)	0.0060 (12)	0.0063 (14)
O12	0.0297 (10)	0.0414 (12)	0.0477 (11)	0.0036 (8)	0.0120 (8)	0.0059 (9)
O13	0.0321 (9)	0.0332 (10)	0.0339 (9)	0.0005 (7)	0.0081 (7)	0.0147 (8)
O14	0.0379 (10)	0.0288 (10)	0.0280 (9)	0.0092 (8)	0.0063 (7)	0.0057 (8)
C15	0.0414 (15)	0.0258 (13)	0.0253 (11)	0.0103 (11)	0.0086 (10)	0.0073 (10)
C16	0.0411 (15)	0.0351 (15)	0.0359 (14)	0.0033 (12)	0.0043 (11)	0.0080 (12)
C17	0.0532 (17)	0.0378 (16)	0.0294 (13)	0.0083 (13)	0.0126 (12)	0.0103 (12)
O18	0.0370 (10)	0.0227 (9)	0.0354 (9)	0.0049 (7)	0.0046 (7)	0.0110 (8)
O19	0.0311 (9)	0.0307 (10)	0.0510 (11)	0.0069 (8)	0.0077 (8)	0.0129 (9)
C21	0.0399 (15)	0.0298 (15)	0.0351 (14)	0.0042 (11)	0.0074 (11)	0.0121 (12)
C22	0.0425 (15)	0.0291 (14)	0.0307 (13)	0.0038 (11)	0.0035 (11)	0.0100 (11)
C23	0.0322 (13)	0.0275 (13)	0.0321 (12)	0.0102 (10)	0.0064 (10)	0.0121 (11)
C24	0.0422 (15)	0.0286 (14)	0.0331 (13)	0.0028 (11)	0.0042 (11)	0.0140 (12)
C25	0.0325 (13)	0.0268 (13)	0.0349 (13)	0.0068 (10)	0.0073 (10)	0.0085 (11)
C26	0.0428 (15)	0.0322 (15)	0.0400 (14)	0.0115 (12)	0.0153 (12)	0.0146 (12)
O27	0.0370 (10)	0.0368 (11)	0.0310 (9)	0.0039 (8)	0.0019 (8)	0.0053 (8)
C28	0.0374 (14)	0.0404 (17)	0.0348 (14)	−0.0020 (12)	0.0015 (11)	0.0120 (13)
C31	0.0525 (19)	0.059 (2)	0.0333 (14)	0.0006 (16)	0.0091 (13)	0.0060 (14)
C30	0.066 (2)	0.049 (2)	0.0388 (16)	−0.0109 (16)	0.0027 (14)	0.0126 (15)
C29	0.0459 (17)	0.065 (2)	0.0492 (17)	0.0050 (16)	−0.0018 (14)	0.0293 (17)
O32	0.0376 (11)	0.0576 (15)	0.0490 (12)	0.0053 (10)	0.0111 (9)	0.0120 (11)
O33	0.0349 (10)	0.0435 (12)	0.0654 (14)	0.0144 (9)	0.0148 (9)	0.0343 (11)
O34	0.0727 (15)	0.0466 (13)	0.0281 (10)	−0.0250 (11)	0.0114 (10)	0.0031 (9)
C35	0.0431 (15)	0.0355 (15)	0.0299 (13)	−0.0060 (12)	0.0042 (11)	0.0125 (12)
C36	0.061 (2)	0.062 (2)	0.0487 (17)	0.0073 (17)	0.0258 (16)	0.0180 (17)
C37	0.067 (2)	0.067 (2)	0.0449 (18)	0.0006 (18)	−0.0012 (16)	0.0313 (18)
O38	0.0384 (10)	0.0290 (10)	0.0301 (9)	0.0022 (8)	0.0085 (7)	0.0097 (8)
O39	0.0481 (11)	0.0262 (9)	0.0302 (9)	0.0090 (8)	0.0087 (8)	0.0096 (8)

### Geometric parameters (Å, °)

**Table:**

C1—C2	1.508 (3)	C21—C22	1.504 (4)
C1—O7	1.344 (3)	C21—O27	1.337 (3)
C1—O12	1.206 (3)	C21—O32	1.210 (4)
C2—C3	1.524 (4)	C22—C23	1.522 (4)
C2—H21	0.977	C22—H221	0.988
C2—H374	0.937	C22—H222	0.971
C3—C4	1.526 (3)	C23—C24	1.522 (4)
C3—O13	1.418 (3)	C23—O33	1.423 (3)
C3—H31	0.940	C23—H231	1.066
C4—C5	1.546 (4)	C24—C25	1.542 (4)
C4—O14	1.445 (3)	C24—O34	1.429 (3)
C4—H41	0.989	C24—H241	0.975
C5—C6	1.504 (4)	C25—C26	1.516 (4)
C5—O18	1.435 (3)	C25—O38	1.439 (3)
C5—H51	0.961	C25—H251	0.992
C6—O19	1.429 (3)	C26—O39	1.427 (3)
C6—H61	0.954	C26—H261	0.988
C6—H62	1.004	C26—H262	0.978
O7—C8	1.469 (3)	O27—C28	1.490 (3)
C8—C9	1.513 (4)	C28—C31	1.520 (4)
C8—C10	1.521 (4)	C28—C30	1.514 (5)
C8—C11	1.521 (4)	C28—C29	1.516 (5)
C9—H91	0.974	C31—H311	0.971
C9—H92	0.985	C31—H312	0.967
C9—H93	0.977	C31—H313	0.962
C10—H101	0.956	C30—H301	0.960
C10—H102	0.974	C30—H302	0.966
C10—H103	0.980	C30—H303	0.964
C11—H111	0.990	C29—H291	0.983
C11—H112	0.970	C29—H292	0.979
C11—H113	0.987	C29—H293	0.973
O13—H49	0.807	O33—H37	0.827
O14—C15	1.420 (3)	O34—C35	1.418 (4)
C15—C16	1.508 (4)	C35—C36	1.499 (5)
C15—C17	1.520 (4)	C35—C37	1.512 (4)
C15—O18	1.432 (3)	C35—O38	1.431 (3)
C16—H161	0.986	C36—H361	0.975
C16—H162	0.970	C36—H362	0.967
C16—H163	0.987	C36—H363	0.959
C17—H171	0.968	C37—H371	0.976
C17—H172	0.965	C37—H372	0.955
C17—H173	0.974	C37—H373	0.967
O19—H22	0.849	O39—H5	0.832
			
C2—C1—O7	110.2 (2)	C22—C21—O27	110.8 (2)
C2—C1—O12	124.6 (2)	C22—C21—O32	124.2 (2)
O7—C1—O12	125.2 (2)	O27—C21—O32	125.0 (3)
C1—C2—C3	112.8 (2)	C21—C22—C23	113.8 (2)
C1—C2—H21	102.3	C21—C22—H221	108.0
C3—C2—H21	110.5	C23—C22—H221	108.2
C1—C2—H374	105.5	C21—C22—H222	111.0
C3—C2—H374	112.2	C23—C22—H222	108.3
H21—C2—H374	112.9	H221—C22—H222	107.4
C2—C3—C4	109.1 (2)	C22—C23—C24	110.0 (2)
C2—C3—O13	111.77 (19)	C22—C23—O33	105.6 (2)
C4—C3—O13	113.4 (2)	C24—C23—O33	113.9 (2)
C2—C3—H31	111.7	C22—C23—H231	106.9
C4—C3—H31	100.9	C24—C23—H231	110.2
O13—C3—H31	109.6	O33—C23—H231	109.8
C3—C4—C5	118.7 (2)	C23—C24—C25	119.8 (2)
C3—C4—O14	107.8 (2)	C23—C24—O34	108.3 (2)
C5—C4—O14	104.06 (19)	C25—C24—O34	104.3 (2)
C3—C4—H41	105.0	C23—C24—H241	106.4
C5—C4—H41	109.2	C25—C24—H241	108.8
O14—C4—H41	112.2	O34—C24—H241	108.9
C4—C5—C6	118.2 (2)	C24—C25—C26	119.6 (2)
C4—C5—O18	103.49 (19)	C24—C25—O38	103.7 (2)
C6—C5—O18	109.7 (2)	C26—C25—O38	108.4 (2)
C4—C5—H51	105.4	C24—C25—H251	108.9
C6—C5—H51	104.5	C26—C25—H251	101.8
O18—C5—H51	116.0	O38—C25—H251	115.1
C5—C6—O19	110.7 (2)	C25—C26—O39	113.7 (2)
C5—C6—H61	107.6	C25—C26—H261	105.0
O19—C6—H61	107.9	O39—C26—H261	108.5
C5—C6—H62	113.0	C25—C26—H262	108.2
O19—C6—H62	112.6	O39—C26—H262	107.9
H61—C6—H62	104.6	H261—C26—H262	113.6
C1—O7—C8	121.33 (19)	C21—O27—C28	121.5 (2)
O7—C8—C9	110.5 (2)	O27—C28—C31	101.9 (2)
O7—C8—C10	109.2 (2)	O27—C28—C30	109.1 (2)
C9—C8—C10	112.6 (2)	C31—C28—C30	111.3 (3)
O7—C8—C11	102.4 (2)	O27—C28—C29	110.2 (3)
C9—C8—C11	110.9 (3)	C31—C28—C29	111.3 (3)
C10—C8—C11	110.7 (3)	C30—C28—C29	112.6 (3)
C8—C9—H91	109.0	C28—C31—H311	108.8
C8—C9—H92	109.5	C28—C31—H312	113.1
H91—C9—H92	112.0	H311—C31—H312	106.9
C8—C9—H93	107.4	C28—C31—H313	111.9
H91—C9—H93	110.2	H311—C31—H313	106.7
H92—C9—H93	108.6	H312—C31—H313	109.1
C8—C10—H101	109.9	C28—C30—H301	108.4
C8—C10—H102	109.8	C28—C30—H302	108.0
H101—C10—H102	108.0	H301—C30—H302	109.9
C8—C10—H103	108.1	C28—C30—H303	109.6
H101—C10—H103	110.7	H301—C30—H303	110.6
H102—C10—H103	110.4	H302—C30—H303	110.2
C8—C11—H111	110.5	C28—C29—H291	108.4
C8—C11—H112	106.6	C28—C29—H292	109.2
H111—C11—H112	111.1	H291—C29—H292	110.0
C8—C11—H113	109.5	C28—C29—H293	108.0
H111—C11—H113	111.5	H291—C29—H293	111.1
H112—C11—H113	107.5	H292—C29—H293	110.0
C3—O13—H49	118.0	C23—O33—H37	102.0
C4—O14—C15	108.28 (18)	C24—O34—C35	110.3 (2)
O14—C15—C16	108.2 (2)	O34—C35—C36	108.2 (3)
O14—C15—C17	111.3 (2)	O34—C35—C37	110.9 (3)
C16—C15—C17	112.9 (2)	C36—C35—C37	113.5 (3)
O14—C15—O18	103.72 (19)	O34—C35—O38	104.4 (2)
C16—C15—O18	109.1 (2)	C36—C35—O38	108.6 (3)
C17—C15—O18	111.1 (2)	C37—C35—O38	110.6 (2)
C15—C16—H161	111.7	C35—C36—H361	109.6
C15—C16—H162	106.1	C35—C36—H362	107.2
H161—C16—H162	111.4	H361—C36—H362	110.4
C15—C16—H163	108.4	C35—C36—H363	109.2
H161—C16—H163	111.4	H361—C36—H363	110.6
H162—C16—H163	107.5	H362—C36—H363	110.0
C15—C17—H171	109.6	C35—C37—H371	109.8
C15—C17—H172	110.2	C35—C37—H372	107.5
H171—C17—H172	107.4	H371—C37—H372	110.8
C15—C17—H173	107.8	C35—C37—H373	109.1
H171—C17—H173	110.7	H371—C37—H373	109.7
H172—C17—H173	111.1	H372—C37—H373	109.9
C5—O18—C15	106.46 (18)	C25—O38—C35	107.8 (2)
C6—O19—H22	113.3	C26—O39—H5	107.4

### Hydrogen-bond geometry (Å, °)

**Table:**

*D*—H···*A*	*D*—H	H···*A*	*D*···*A*	*D*—H···*A*
C2—H21···O34^i^	0.98	2.46	3.407 (4)	164
C3—H31···O12^ii^	0.94	2.54	3.442 (4)	161
C9—H91···O39^iii^	0.97	2.60	3.502 (4)	154
C10—H103···O12	0.98	2.40	3.031 (4)	121
C22—H222···O14^iv^	0.97	2.50	3.349 (4)	147
C30—H302···O32	0.97	2.40	3.009 (4)	121
O19—H22···O38^v^	0.85	2.03	2.861 (4)	165
O33—H37···C26	0.83	2.57	3.231 (4)	138
O33—H37···O39	0.83	1.88	2.703 (4)	171
O13—H49···O19^iii^	0.81	2.06	2.842 (4)	162
O39—H5···O18^vi^	0.83	1.98	2.771 (4)	157

Symmetry codes: (i) *x*−1, *y*, *z*−1; (ii) *x*+1, *y*, *z*; (iii) *x*−1, *y*, *z*; (iv) *x*+1, *y*, *z*+1; (v) *x*−1, *y*−1, *z*−1; (vi) *x*+1, *y*+1, *z*+1.
